# Appendicitis and Peritonitis in Children with a Ventriculo-Peritoneal Shunt

**DOI:** 10.3390/children10030571

**Published:** 2023-03-17

**Authors:** Glenn M. C. Fröschle, Johanna Hagens, Philip Mannweiler, Friederike Sophie Groth, Gertrud Kammler, Konrad Reinshagen, Christian Tomuschat

**Affiliations:** 1 Department of Pediatric Surgery, University Medical Center Hamburg-Eppendorf, 20246 Hamburg, Germany; 2 Central Controlling Division, University Medical Center Hamburg-Eppendorf, 20246 Hamburg, Germany; 3 Department of Neurosurgery, University Medical Center Hamburg-Eppendorf, 20246 Hamburg, Germany

**Keywords:** ventriculoperitoneal shunt, children, appendicitis, surgical management

## Abstract

The purpose of this study was to outline the management of patients with appendicitis and ventriculoperitoneal shunt (VPS) in the largest pediatric surgery department in Germany. Patients with VPS presenting with an acute abdomen between 2012 and 2022 at a tertiary-care pediatric facility were the subject of a retrospective descriptive analysis. Patients were divided into two groups based on their diagnoses: group A (appendicitis) and group B (primary peritonitis). Medical records were analyzed to look at the diagnostics, operative approach, complications, peritoneal and liquor culture, and antibiotic treatment. A total of seventeen patients were examined: seven patients in group A and ten individuals in group B. In the present study patients in group A typically presented younger, sicker, and with more neurological symptoms than those in group B. All patients with appendicitis had their VPS exteriorized, and a new shunt system into the peritoneum was reimplanted 20 days later. Surgery should be aggressively administered to patients who present with an acute abdomen and a VPS. Change of the whole shunt system is suggested. Shunt infection and dysfunction should be ruled out in patients with abdominal symptoms, and surgical care should be started with a low threshold.

## 1. Introduction

Abdominal-shunt-related problems are known in patients with ventriculoperitoneal shunts (VPS). Clinically, it can be difficult to set the right diagnosis when a child with a VPS experiences fever, anorexia, nausea, vomiting, acute abdominal pain, and right lower quadrant peritoneal signs, in addition to an elevated white blood cell count. When a child with a VPS presents with clinical symptoms of an “acute abdomen”, physicians sometimes face a clinical challenge, as a primary intra-abdominal and shunt-independent disease must be distinguished from shunt-related infection [1]. Appendicitis, primary peritonitis, adhesive small bowel obstruction, or an infected abdominal pseudocyst are usually included in the differential diagnosis [2]. Furthermore, many shunt-treated patients have urological and/or abdominal comorbidities that predispose them to urological infections or bowel perforation, in addition to usual sources of peritonitis such as a ruptured appendix [3]. The best way to set the diagnosis and to manage a VPS during an acute abdominal or pelvic infection, however, remains debatable. There are only a limited number of cases reported in the literature of children with VPS being managed for appendicitis. The details of each report are very heterogenous, making it difficult to draw clear conclusions. Each of the studies have limitations. The rarity of these cases results in reports spanning decades, during which time many clinical factors are almost certain to have substantially differed [4].

The goal of the current study was to identify potential risk factors so that patients who may have appendicitis might be identified from those with peritonitis so that, in addition to other reasons, they can be appropriately referred for treatment to stop further damage.

## 2. Materials and Methods

### 2.1. Patient Identification

The study followed the principles of the Helsinki Declaration and was approved by the local institutional review board of the Hamburg ethics committee (2022-300165-WF, 23 February 2022). From June 2012 to May 2022, all children (age limit 18) with VPS who were admitted for abdominal pain, fever, peritonism and/or presented with signs of an acute abdomen had their hospital records from the University Medical Center Hamburg-Eppendorf (UKE) Pediatric Surgery Department reviewed and were included in the study. The appropriate ICD-10 (International Statistical Classification of Diseases and Related Health Problems) codes for appendicitis, peritonitis, and VPS from the DRG-System (Diagnosis related group) were also added to the search algorithm. Patients aged over 18 and with a history of appendectomy were excluded. In total, 17 patients met the inclusion criteria.

### 2.2. Study Protocol

The medical patient records were retrospectively analyzed to look at the presentation, diagnostic studies, operative approach (laparoscopic, primary open, conversion to open), complications, shunt exteriorization, peritoneal culture, liquor culture and length of the antibiotic treatment. In addition, information on length of stay (LOS) and long-term outcome was acquired. The purpose of the study was to describe the characteristics of patients with VPS and their initial clinical symptoms. Additionally, the postoperative course and perioperative morbidity were recorded. The primary endpoint was the clinical spectrum, which included the onset of symptoms such as abdominal pain, neurological symptoms, elevated inflammatory parameters (CRP, leukocytes), and the intraoperative findings. Secondary endpoints included LOS, shunt exteriorization, and re-implantation in accordance with the prescribed antibiotic regime (duration).

### 2.3. Follow-Up

Regular outpatient clinic visits were used for the collection of follow-up data until the age of 18. Patients with primary exteriorization and who had their appendix removed were monitored every six months on a weekly basis after they had initially been seen to monitor for early abdominal complications. Following pre-implantation of a peritoneal catheter, patients first visited the neurosurgical outpatient clinic on a regular basis. The frequency of visits depended greatly on the patient’s age and was determined by several protocols.

### 2.4. Statistical Analysis

Patient demographics were calculated as median with interquartile range, as well as maximum and minimum values given as total range. Statistical analysis was performed descriptively using Microsoft Office Excel (version 16.56, Microsoft, Seattle, WA, USA) and GraphPadPrism (version 9.2.0, GraphPad, San Diego, CA, USA). For testing normality of data distribution, we used the Shapiro–Wilk test. Differences between both study groups were analyzed by using the Welsh-T test. A *p*-value < 0.05 was suggested as significant.

### 2.5. Diagnostic Algorithm

In patients with abdominal pain and a ventricular peritoneal shunt, a comprehensive and individualized approach is required. For all patients, a detailed medical history and physical examination was performed, along with blood work, including a complete blood count, electrolyte panel, liver function tests, and inflammatory markers. An ultrasound of the abdomen (US) was almost always obtained to evaluate for any structural abnormalities or signs of inflammation or infection. If necessary, a conventional abdominal X-ray (AXR) or computed tomography scan (CT) can be done. Additional testing or referral to a specialist may be necessary, depending on the results of the initial workup. However, the diagnostic approach should always be tailored to the specific needs and circumstances of the patient, considering potential risks and benefits of any diagnostic tests or interventions, as well as the patient’s overall health and well-being.

## 3. Results

**Figure 1:**
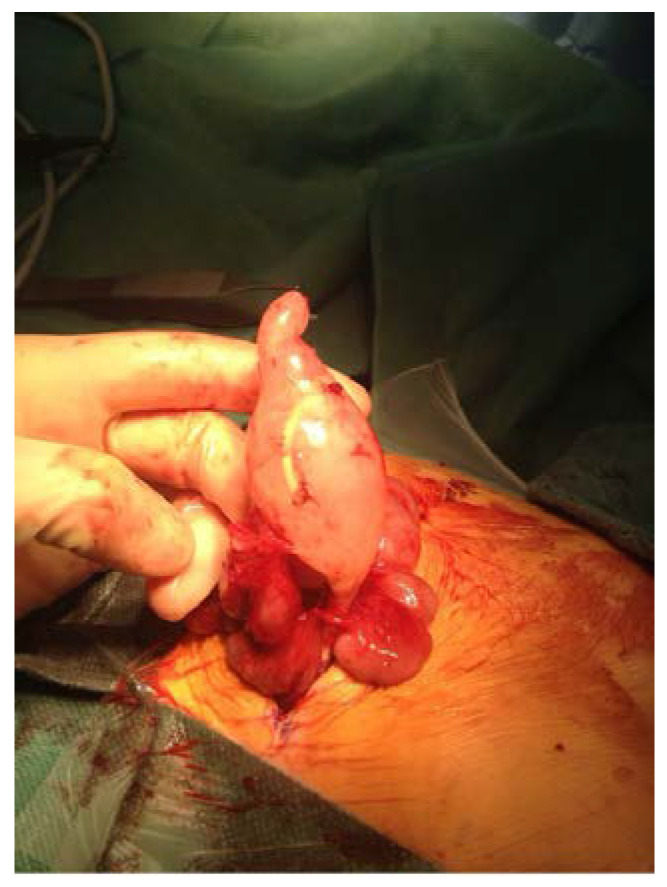
Infected pseudocyst in a patient with VPS and acute appendicitis.

**Figure 2:**
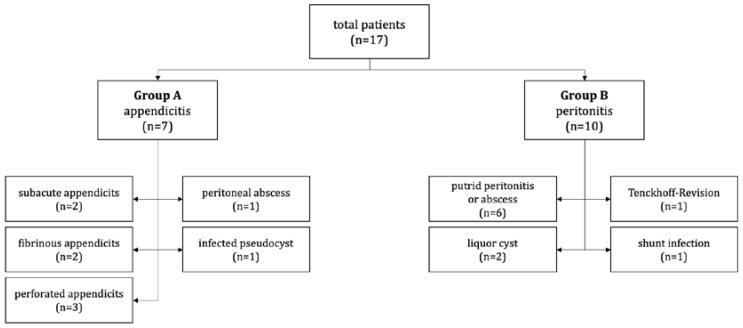
Overview of the surgical findings and reasons for acute abdomen in the patients included in this study.

We identified 17 patients with VPS who reported to the emergency room between June 2012 and May 2022 with acute abdominal discomfort or indications of an acute abdomen. Out of those, seven patients (group A) had their appendix removed, and those cases were considered as appendicitis, which was confirmed by histopathology; two cases were subacute (Grade 1), two were fibrinous (Grade 3), and three were perforated (Grade 4) [5]. Additionally, one child had a huge peritoneal abscess brought on by an inflamed appendix, while another had an infected pseudocyst (Figure 1). In the primary peritonitis group (group B) we identified ten patients. Five presented with putrid peritonitis or an abscess without focus. Another two had a pseudocyst, one needed a dialysis catheter revision, and one had a shunt infection (Figure 2).

### 3.1. Group A: Appendicitis (n = 7)

The patients in this cohort had a median age at surgery of 34 months (with a range of 7 to 195 months) and a mean age of 53 months. The main patient characteristics are summarized in Table 1. Six patients (85.7%) were females and one (14.3%) was male. Table 2 provides a description of the hydrocephalus etiologies. The length of time from the onset of symptoms to the start of surgery was an average of 3 days (1–10 days). Abdominal pain accounted for the most common symptom, and was seen in six (85.7%) of the patients, followed by a distended abdomen in four (57.1%), vomiting in four (57.1%), headache in two (28.6%), and diarrhea in one (14.3%) patient. Additionally, three individuals (42.9%) presented with seizures, and three more (42.9%) displayed symptoms of increased intracranial pressure (ICP). From an ultrasound, free fluid was found in five patients (71.4%), septate fluid collections in three (42.9%), hyperperfusion of the intestine in two (28.6%), fat imbibition and thickening of the intestinal wall in two (28.6%) and adhesions in one (14.3. In six (85.0%) patients, the abdominal cultures were positive for *Staphylococcus epidermidis*, *Citrobacter koseri*, *Streptococcus constellatus*, *Escherichia coli*, *Enterococcus faecalis*, *Bacterioides fragiles* and *Staphylococcus aureus*, respectively. Antibiotics used during perioperative patient treatment are summarized in Table 3. *Staphylococcus epidermidis*, *Staphylococcus aureus*, and *Enterococcus faecalis* were the most prevalent bacteria found in the cerebrospinal fluid (CSF) of five (71.4%) individuals. For most patients, the antibiotic therapy lasted 14 days. All patients (100%) had their catheter exteriorized at the thoracic level initially at the primary operation. For group A, the median follow-up period after surgery was 55 months (32–106 months). Within the first year following the appendectomy, two patients (28.6%) underwent a second shunt revision operation, with one (14.3%) due to proximal failure and one (14.3%) due to a malfunctioning distal catheter. One patient from the subgroup with a positive CSF culture experienced shunt infection. Within the first year following the appendectomy, four patients (40.0%) underwent a second shunt revision operation, with three (30.0%) due to a malfunctioning distal catheter and one due to proximal failure (10.0%). One patient from the subgroup with a positive CSF culture experienced shunt infection. During the time of follow-up, no patient passed away.

### 3.2. Group B: Peritonitis (n = 10)

The patients in this cohort had a median age at surgery of 71 months (ranging 9–180 months) and a mean age of 93 months. The main patient characteristics are summarized in Table 1. Nine (90.0%) had a distended abdomen on presentation, followed by abdominal pain in eight individuals (80.0%). Vomiting was seen in four (40.0%), headache in two (20.0%), diarrhea in two (20.0%) and obstipation in two (20.0%). Additionally, two patients (20.0%) presented with seizures, and one (10.0%) displayed symptoms of ICP. In an ultrasound, septate fluid was found in six patients (60.0%), free fluid collections in five (50.0%), hyperperfusion of the intestine in four (40.0%), fat imbibition and thickening of the intestinal wall in one (10.0%) and signs of an ileus in two (20.0%). In four (40%), the abdominal cultures were positive for *Staphylococcus epidermidis*, *Serratia marcensens*, *Escherichia coli*, *Enterococcus faecalis* and *Pseudomonas*. Antibiotics used during the perioperative course are summarized in Table 3. *Staphylococcus epidermidis*, *Streptococcus oralis* and *salivarius*, *Enterococcus*, *Enterobacter cloacae*, *Staphylococcus warneri* were the most prevalent bacteria found in the CSF of four (40.0%) individuals. For most patients, antibiotic therapy lasted 14 days. The shunt was not externalized in two patients of group B: One child only had a revision of the shunt, with new placement in the upper abdomen. Another child from this group did not have surgery—the peritonitis was treated conservatively with antibiotics. Additionally, one child with VPS received a Tenckhoff catheter revision and had their shunt changed to another system intraoperatively and therefore had zero days to shunt reimplantation. All distal catheters were reinserted using a laparoscopic-assisted or open method into the peritoneal cavity. The median follow-up period after surgery was 55 months (13–82 months). During the time of follow-up, one patient passed away.

## 4. Discussion

In the present cohort of children with VPS, 17 patients presented with an acute abdomen at the University Medical Center Hamburg-Eppendorf (Kinder UKE) between 2012 and 2022. Given that we implant 35 VPSs per year (10 years/350 patients), we report an abdominal complication rate of 4.8% for the current cohort. Interestingly, the children included in the study were younger than the typical appendicitis patient. The pediatric surgery community is aware that toddlers and very young children are frequently neglected when it comes to appendicitis; as a result, the perforation rate is higher, and the clinical course is more severe in these patients [6]. In 58% of the patients in group A, the appendix was, however, only minimally inflamed and lacked severe forms of infections, such as gangrenous changes. As a result, there is a chance that the appendicitis was more of a co-reaction to the shunt infection than anything else. This is especially possible given the young age of the patients in this cohort. Shunt infections are most common in the first year of life, occurring anywhere between 4% and 20% of the time, and declining to 3–5% the following year [7]. Up until it is proven differently, shunt infection should be suspected, particularly when neurological symptoms are present. The onset of new neurological symptoms in a child with VPS is always indicative of a shunt dysfunction [8]. Therefore, a distal malfunction brought on by obstruction of the catheter’s distal tip should be ruled out, in addition to a proximal dysfunction, which is by far the most frequent cause of shunt failure [9]. After confirmation of elevated intracranial pressure (ICP) and/or ventricular enlargement in a CT scan or MRI, immediate shunt system removal is advised due to the patient’s acute symptoms, which also can lead to a quick diagnosis [10]. On the other hand, the median time from the onset of symptoms to operation or final diagnosis in the present cohort was 3 days for group A and 4.5 days for group B, making it simple to understand that often a “diagnostic loop” may delay the proper diagnosis, which underlines the need for a protocol regarding quick diagnosis.

Generally, CT is a quick and accessible modality that can promptly identify any alteration in ventricular size. It can also detect any potential shunt system obstructions [3]. When a quick diagnosis is necessary in an emergency, CT is especially helpful [11]. On the other hand, MRI offers superior anatomical detail and can spot changes in the peritoneal cavity as well as ventricular size alterations. It is especially helpful in identifying minute alterations in the shunt system that might not be seen in CT scans [12]. The clinical presentation, the need for a quick diagnosis, and the imaging center’s experience all influence the modality choice [13]. To diagnose the abdominal portion of a VPS, imaging modalities such as abdominal X-ray (AXR), ultrasonography (US), or CT can be used. The easiest and most common method for evaluating the abdominal part of a VP shunt is an abdominal AXR. It can identify the location of the shunt tube as well as any disconnections or kinks. AXR, however, are unable to give precise information on the organs or structures found within the abdomen [14]. Another imaging technique that can be utilized to assess the abdominal component of a VP shunt is US [1]. It is especially helpful in identifying any abdominal fluid accumulation or ascites that can point to a shunt issue. Young children benefit greatly from US because it is non-invasive, doesn’t involve ionizing radiation, and is safe. In contrast, the abdominal part of a VP shunt can also be thoroughly evaluated with CT. [2,10]. Since almost all patients with VPS have free fluid collections in the pelvis, which happens in patients with advanced perforated appendicitis as well, difficulties typically develop owing to the detection of free fluid, septate fluid collections, or cyst-like structures in the abdomen in those patients [15]. However, free fluid or septate fluid collections are not always suggestive of an urgent abdominal issue because an abdominal pseudocyst (APC) could potentially be present. This is especially true if the child has no neurological symptoms suggestive of shunt dysfunction. The incidence of APCs ranges from 0.7% to 4.5% [3]. APCs, surrounded by thickened peritoneal serosa, prevents CSF resorption, which may result in shunt malfunction; they tend to develop weeks to years after the placement of the shunt, and it is believed that they are caused by local inflammatory responses connected to an infectious process in the CSF that produce adhesions. Other mechanisms are connected to repeated surgical treatments. We recommend a laparotomy and/or percutaneous draining as cyst removal procedures because we believe that every APC is infectious, even though surgical evacuation may not always be necessary [3,15,16].

There was no significant difference in the preoperative laboratory results among our cohort, which might have affected the ability to recognize patients with acute appendicitis.

However, even if no infection in the CSF can be confirmed, a new distal catheter can be inserted in another location of the abdomen after the shunt has temporally exteriorized.

Shunt duration was defined as the interval between the last shunt implanted and the onset of acute abdominal symptoms: this was similar in both groups and was 4 and 5 months, respectively. According to the literature, most shunt infections tend to develop within three months of their implantation [3,7,16]. Successive shunts (revisions) have been found to have progressively higher infection rates [16]. Young age, female sex, public insurance, intraventricular hemorrhage, respiratory complex chronic condition, hospital volume, and surgeon case volume are additional characteristics that are significantly linked with infection [17]. According to the published literature, VPS infection rate ranges from 3% to 20% [7]. Numerous investigations have revealed that *S. aureus* and coagulase-negative staphylococci are the two main pathogens in shunt infections [16]. However, according to Wang et al. [18], Gram-negative bacteria are also responsible for 7% to 24% of all VPS infections, without having an obvious abdominal focus. Therefore, it is not surprising that we also found Gram-negative bacteria in group B, although less often (40%) compared to group A (85%). Group A had a greater variety of the identified microorganisms than group B.

We had a CSF culture positivity rate of 72% in group A and 40% in group B, which may not come as a surprise, given that a primary abdominal focus might also result in ascending infections. In fact, patients with perforated appendicitis have been shown to have a higher risk of ascending infection than those with non-perforated appendicitis [4]. An operation with an exteriorization of the shunt should avoid contact with the suspected perforation [3]. However, research indicates that non-perforated appendicitis patients have a minimal risk of ascending shunt infection [4]. Because the catheter is exposed to the contaminant for a shorter period when a patient has acute peritonitis, the chance of an ascending infection is probably lower [3]. CSF cultures taken during a laparotomy from the shunt valve or straight from the distal catheter can be utilized to conclusively identify an ascending (or descending) shunt infection. The findings should be known before installing a new shunt device. The complete shunt system should be removed if the CSF culture is positive, and an external ventricular drain (EVD) should be implanted until such time that the infection is resolved [8].

The average length of the prescribed antibiotic course is 14 days, and it typically consists of the following antibiotics: amoxicillin/clavulanate, ampicillin/sulbactam, meropenem, piperacillin/tazobactam, and vancomycin [3,7,10,15] (Table 3). However, the decision to prescribe an empiric antibiotic regimen should be determined on a case-by-case basis in accordance with local protocols and in consultation with a microbiologist or infectious diseases specialist because there is not enough data to do so otherwise [4]. Generally speaking, the CSF findings must be benign from a microbiological and laboratory chemical standpoint in the case of inflammatory CSF syndrome.

Based on this, the therapy’s length can be determined. However, it is controversial whether the catheter’s distal end should be reinserted into the peritoneum or implanted preferably into the atrium of the heart to prevent a second abdominal approach. In our case series, each system was completely changed after suspected infection and reinserted into the peritoneal cavity. We had a moderate revision rate of the whole shunt system within one year (group A 28.6% vs. group B 40.0%) in both groups, although it is questionable and impossible to prove in the current study whether reinsertion into the abdominal cavity was related to consecutive revision surgery.

## 5. Limitations

Due to the retrospective nature of our study and changes in practice, it has limitations. Despite being retrospective, our study was able to examine a sizable study group in a condition with a very low incidence. This study is also constrained by its retrospective design and the fact that only one center was used for data collection. As a result, the recommendations made in this study are not backed up by solid data. To create management recommendations that are supported by evidence, additional research is required. However, these recommendations might be helpful in the regular management of these individuals.

## 6. Conclusions

Our findings suggest that aggressive surgical treatment should be offered to patients who present with an acute abdomen and a VPS, even though careful consideration should be made based on the clinical and microbiological findings. It is advised that the entire shunt system be changed. Despite the absence of obvious appendicitis symptoms, it is challenging to determine whether the appendix should be removed; however, given that these conditions often have a very complicated course, an appendectomy would be justified. To ensure a secure reimplantation into the peritoneal cavity, the course of antibiotics should extend to at least 14 days.

## Figures and Tables

**Table 1:** Patient characteristics. BMI (Body mass index), CRP (C-reactive protein), IQR (inter quartile range).

	Group A (*n* = 7) Appendicitis	IQR	Group B (*n* = 10) Peritonitis	IQR	*p*
male (*n*)	1 (14.3%)		5 (50.0%)		-
female (*n*)	6 (85.7%)		5 (50.0%)		-
age at operation (m)	34.0 (7.0–195.0)	17.0–47.0	71.0 (9.0–180.0)	31.0–163.0	0.2489
height (cm)	87.0 (59.0–168.0)	71.8–127.5	100.0 (61.0–163.0)	79.5–138.0	0.6891
weight (kg)	11.1 (5.5–82.0)	8.4–43.8	17.0 (5.8–63.0)	9.0–52.5	0.8866
BMI	15.5 (12.4–29.0)	13.5–22.8	15.6 (12.0–23.7)	12.6–21.7	0.8568
preoperative CRP (mg/dL)	152.0 (25.0–363.0)	123.0–327.0	104.0 (0.0–305.0)	16.3–256.3	0.3551
temperature (°C)	39.0 (37.7–40.7)	38.0–40.2	38.4 (36.7–39.0)	37.3–38.7	0.0925
leucocytes (x 10^9^/l)	13.9 (2.5–27.2)	11.0–22.7	19.5 (7.1–29.5)	12.0–25.2	0.3622
age at first shunt placement (m)	7.5 (0.0–194.0)	1.5–67.3	4.5 (2.0–150.0)	2.0–45.0	0.7960
age at last shunt placement (m)	17.5 (6.0–194.0)	6.8–77.0	20.0 (6.0–150.0)	8.0–108.0	0.9499
shunt duration (m)	4.0 (0.0–40.0)	0.8–15.3	5.0 (0.0–64.0)	0.3–18.8	0.6992
operation time (min)	123.0 (73.0–200.0)	93.0–166.0	97.0 (38.0–290.0)	53.0–162.5	0.6707
shunt externalization (*n*)	7 (100.0%)		8 (80.0%)		
time to shunt reimplantation (d)	20.0 (3.0–28.0)	8.5–27.5	14.0 (0.0–25.0)	7.0–15.0	0.3316
hospital length of stay (d)	25.0 (11.0–40.0)	18.0–36.0	26.0 (4.0–62.0)	18.3–42.3	0.6072

**Table 2:** Reason for initial shunt placement in the study’s patients.

	Group A (*n* = 7)	Group B (*n* = 10)
Post-hemorrhagic Hydrocephalus	3	5
Hydrocephalus (no specification)	1	2
Hydrocephalus at birth	0	2
Hydrocephalus by tumor	1	1
Hydrocephalus by malformation	1	0
Hydrocephalus occlusus	1	0

**Table 3:** Antibiotics used during perioperative patient treatment.

	Group A (*n* = 7)	Group B (*n* = 10)
Nitroimidazole		
Metronidazole	7	3
Glycopeptide		
Vancomycin	4	6
Teicoplanin	0	1
Cephalosporine		
Cefuroxime	4	2
Cefotaxime	2	2
Ceftriaxone	1	1
Cefazoline	0	1
Ceftazidime	0	1
Carbapenems		
Meropenem	1	3
Penicillin		
Ampicillin/Sulbactam	2	1
Piperacillin/Tazobactam	0	2
Penicillin G	0	1
Flucloxacillin	1	0
Aminoglycoside		
Tobramycin	2	2
Macrolide		
Erythromycin	2	0
Fluocinolone		
Ciprofloxacin	0	1
Sulphonamide		
Cotrimoxazole	1	0

## Data Availability

The data described in this study are accessible from the corresponding author upon request. Due to ethical and private constraints, the data are not publicly available.
